# Treacle Sticks the Nucleolar Responses to DNA Damage Together

**DOI:** 10.3389/fcell.2022.892006

**Published:** 2022-05-12

**Authors:** Zita Gál, Blanca Nieto, Stavroula Boukoura, Anna Vestergaard Rasmussen, Dorthe Helena Larsen

**Affiliations:** Nucleolar Stress and Disease Group, Danish Cancer Society Research Center, Copenhagen, Denmark

**Keywords:** DNA damage response, DNA repair, chromatin, n-DDR, nucleolus, rDNA, treacle/TCOF1, Treacher Collins syndrome

## Abstract

The importance of chromatin environment for DNA repair has gained increasing recognition in recent years. The nucleolus is the largest sub-compartment within the nucleus: it has distinct biophysical properties, selective protein retention, and houses the specialized ribosomal RNA genes (collectively referred to as rDNA) with a unique chromatin composition. These genes have high transcriptional activity and a repetitive nature, making them susceptible to DNA damage and resulting in the highest frequency of rearrangements across the genome. A distinct DNA damage response (DDR) secures the fidelity of this genomic region, the so-called nucleolar DDR (n-DDR). The composition of the n-DDR reflects the characteristics of nucleolar chromatin with the nucleolar protein Treacle (also referred to as TCOF1) as a central coordinator retaining several well-characterized DDR proteins in the nucleolus. In this review, we bring together data on the structure of Treacle, its known functions in ribosome biogenesis, and its involvement in multiple branches of the n-DDR to discuss their interconnection. Furthermore, we discuss how the functions of Treacle in ribosome biogenesis and in the n-DDR may contribute to Treacher Collins Syndrome, a disease caused by mutations in Treacle. Finally, we outline outstanding questions that need to be addressed for a more comprehensive understanding of Treacle, the n-DDR, and the coordination of ribosome biogenesis and DNA repair.

## 1 Introduction

Eukaryotic nuclei contain several sub-compartments, the largest being the nucleolus. The core function of the nucleolus is to produce ribosomes enabling protein production. This cellular function is preserved across all domains of life, from archaea to humans and is a prerequisite for growth and proliferation (Petrov et al., 2014). The production of ribosomes, also known as ribosome biogenesis, is initiated by transcription of the ribosomal RNAs (rRNAs) that associate with ribosomal proteins to form ribosomal subunits. rRNA transcription and assembly of pre-ribosomal subunits occur in the nucleolus, with subsequent export to the cytoplasm, where protein translation takes place (Baßler and Hurt, 2019).

Both ribosomal RNAs and the proteins involved in ribosome biogenesis are highly concentrated in the nucleolus, creating a unique biophysical environment that enables the nucleolus to exist as a distinct body in the absence of a membrane due to liquid-liquid phase separation processes (Mangan et al., 2017). The high concentration of ribosomal RNAs comes from transcription of the ribosomal RNA genes, also referred to as the rDNA, which localizes in the nucleolus. Human cells have been estimated to have 3–400 copies of ribosomal RNA genes arranged in clusters on the five acrocentric chromosomes (Schmickel, 1973; Sakai et al., 1995). These genes account for up to 60% of total cellular transcription in eucaryotic cells and ensure that the translational capacity meets the demand for protein production (Zylber and Penman, 1971; Warner, 1999).

rDNA has one of the highest recombination frequencies observed in healthy individuals and this is further increased in cancer patients (Stults et al., 2008, 2009). Pronounced variation is found in different clusters, between individuals but also as a result of meiotic recombination (Stults et al., 2008). Such variation can arise from high level of transcriptional activity interfering with replication; either directly through collision between the transcription and replication machinery (García-Muse and Aguilera, 2016), or indirectly through formation of secondary structures such as R-loops. Both processes can result in DNA double-strand breaks (DSBs), a very harmful type of DNA lesion, and potentially compromise genome integrity (Crossley et al., 2019). In further support of transcription being linked to rDNA instability, rDNA DSBs were found to occur non-randomly and overlap with active chromatin marks (H3K4me3) (Tchurikov et al., 2015). Furthermore, the large number of identical rDNA sequences located in close proximity can also pose a threat to genome integrity through faulty intra-chromosomal or inter-chromosomal recombination that can lead to both copy number variation and large-scale rearrangements (Potapova et al., 2019). Finally, rDNA features, such as the high GC-content, can facilitate G-quadruplex formation, interfere with replication and cause DNA DSBs (Wallgren et al., 2016; Wang et al., 2019; Linke et al., 2021).

To maintain genome stability and counteract the potential detrimental consequences of DSBs, cells have a complex network of signalling pathways referred to as the DNA damage response (DDR) that detects aberrant DNA structures and modifies the surrounding chromatin in order to promote DNA repair (Jackson and Bartek, 2009). The DDR also signals globally in the cell to activate cell cycle checkpoints and delay cell cycle progression to limit duplication or segregation of damaged DNA. The DDR is driven by the DNA-damage kinases Ataxia telangiectasia mutated (ATM), Ataxia telangiectasia and Rad3-related (ATR) and DNA-dependent protein kinase (DNA-PK), that play essential roles both at the break site, phosphorylating chromatin components and DDR factors, and globally where phosphorylation cascades drive checkpoint activation and regulate transcription and replication (Blackford and Jackson, 2017).

Investigations conducted over the last few decades have driven significant advances in our understanding of the DDR, but have also revealed new levels of complexity that call for further investigations. Recently, we have come to appreciate that the DDR takes different forms depending on the chromatin context and the physical location in which the damage occurs (Mitrentsi et al., 2020). In this context, the nucleolus stands out as an organelle of particular interest: its physical organization leads to selective retention of proteins, and its chromatin composition is not found elsewhere in the cell. In agreement with this, recent studies have characterized specialized mechanisms operating in the nucleolus in response to rDNA damage, previously referred to as the nucleolar DDR (n-DDR) recently reviewed in Korsholm et al., 2020. In brief, these mechanisms inhibit nucleolar transcription and either facilitate rapid rDNA repair or, upon persistent DNA damage, lead to reorganization of the nucleolus promoting homology-dependent repair (Kruhlak et al., 2007; Harding et al., 2015; van Sluis et al., 2016; Warmerdam et al., 2016; Korsholm et al., 2019; Marnef et al., 2019; Mooser et al., 2020).

At the mechanistic level, one of the striking differences between the canonical DDR and the n-DDR is the role of the nucleolar protein Treacle. Treacle is a well-characterized ribosome biogenesis factor, that promotes rRNA transcription and processing, but it has only recently been implicated in DNA repair (Dixon et al., 2007; Ciccia et al., 2014; Larsen et al., 2014). In the n-DDR Treacle is emerging as a central coordinator of responses to DNA damage. The processes coordinated by Treacle include transcriptional inhibition, DSB repair, replication stress, oxidative stress, osmotic stress, and R-loop formation, suggesting that Treacle functions as a hub that facilitates the nucleolar stress responses.

In this article, we review the data on Treacle’s function in ribosome biogenesis and compare it to its role in maintenance of rDNA. We also discuss the potential underlying causes of Treacher Collins Syndrome, a rare disease caused by mutations in Treacle, including the emerging understanding of Treacle as a coordinator of nucleolar stress responses.

## 2 Treacle: A Ribosome Biogenesis Factor

### 2.1 Discovery of TCOF1 and Treacher Collins Syndrome

Treacle is the protein encoded by the TCOF1 gene, which is predominantly mutated in Treacher Collins Syndrome (TCS). TCS is a rare autosomal dominant disorder present in 1/50,000 births (Fazen et al., 1967) that was first described in 1900. The TCS phenotype is characterized by abnormalities that affect the head and neck, such as malformation of the external and middle ears, which results in bilateral conductive hearing loss (Phelps et al., 1981), cleft palate, hypoplasia of the facial bones, and lateral downward slanting of the lower eyelids (Rovin et al., 1964; Fazen et al., 1967). It was, however, not until 1996 that the TCOF1 gene was identified by positional cloning by the Treacher Collins Collaborative Group (The Treacher Collins Syndrome Colla et al., 1996). More than 150 different mutations have been identified so far with exon 23 and 24 representing a hotspot (approximately 30% of all mutations). In a study with 146 TCS patients, 92% of the patients having the TCOF1 gene affected presented frameshift mutations that resulted in a premature termination codon. The remaining 8% presented intragenic or large microdeletions, also resulting in TCOF1 haploinsufficiency (Edwards et al., 1997; Vincent et al., 2016).

The identification of causative mutations in the TCOF1 gene (85% of the cases (Vincent et al., 2016)), but also in the RNA polymerase I (Pol I) subunits POLR1C, POLR1D and recently, POLR1B (Dauwerse et al., 2011; Sanchez et al., 2020) strongly suggests that perturbation of ribosome biogenesis is the underlying cause of Treacher Collins Syndrome.

### 2.2 Treacle Structure

**FIGURE 1:**
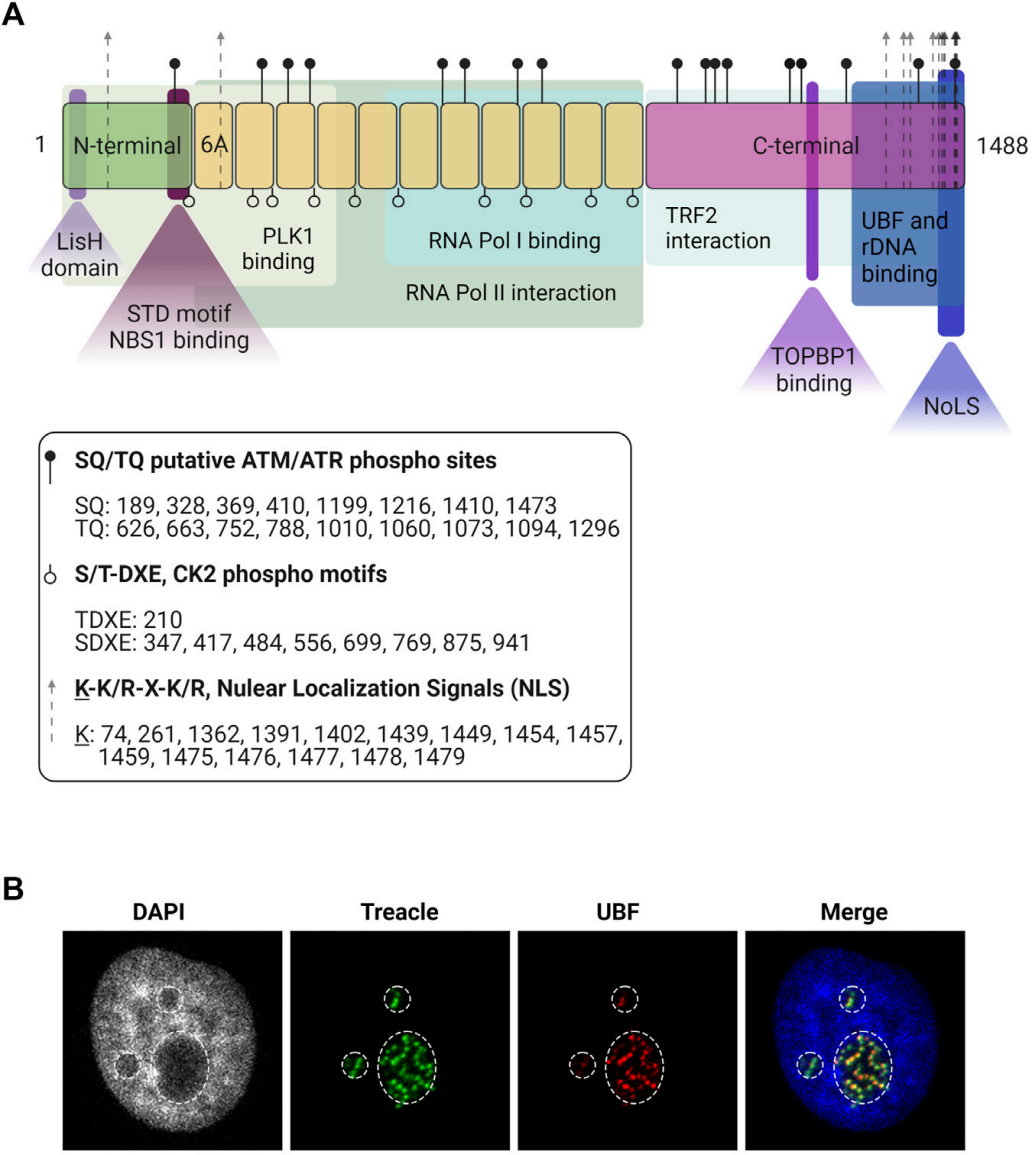
Treacle structure and cellular localization. **(A)** The structure of the most common isoform of Treacle. Treacle isoform d (1488 amino acids, 152 kDa, NP_001128715.1) is encoded by the Treacle transcript variant 4, containing 27 exons. It is an intrinsically disordered protein with N- (exons 1–6, aa 1–213) and C-terminal (exons 17–26, aa 954–1,488) regions, and 11 central repeat domains (exons 6A–16, aa 214–953). The N-terminal harbors a LisH domain (aa 6–38), and contains a nuclear localization signal (NLS, aa 74–77). The SDT-like motifs SETE (aa 171–174), SEDT (200–203), SDET (207–210), are responsible for NBS1 binding, and PLK interaction site was also mapped to the N-terminal domain. The 11 central repeats contain several CK2 and ATM/ATR phosphorylation sites. The central region is responsible for Pol II interaction, and aa 526–953 mediates Pol I binding. Repeat domain 6A furthermore contains a NLS but 6A is not present in all isoforms. The C-terminal region is important for Treacle localization, it contains several potential NLSs (aa 1,362–1,482). The last 41 aa of the protein mediates nucleolar localization. The C-terminal furthermore mediates rDNA and UBF binding (aa 1,294–1,488). It contains several SQ/TQ phospho motifs, three serines (S1227, S1228, S1236) responsible for TOPBP1 recruitment, and a TRF2 interaction site. Created with BioRender.com. **(B)** Immunofluorescence images of Treacle and UBF in the dense fibrillar component, a sub-compartment of the nucleolus where newly transcribed rRNA is located. The nucleolar area is outlined based on lower intensity of the DNA (DAPI) staining.

The most abundant splicing variant of the TCOF1 gene is a 152 kDa nucleolar protein derived from 27 exons (So et al., 2004). The TCOF1 gene product is a low-complexity protein, with unique N-terminal and C-terminal domains separated by a region of repeated motifs (Wise et al., 1997) (Figure 1A). The N-terminal contains a LisH domain, thought to promote dimerization. The C-terminal region presents high lysine content and it is essential for correct localization: it contains nuclear localization signals (NLSs) required for entering the nucleoplasm and a nucleolar localization signal (NoLS) located in the last 41 amino acids of the protein (Marsh et al., 1998; Winokur and Shiang, 1998).

Treacle is a highly phosphorylated protein with numerous consensus Casein Kinase phosphorylation sites, where Casein Kinase 2 (CK2) is responsible for phosphorylation. In its central region, composed of 11 repeated units of acidic residues, separated by stretches of basic amino acids, Treacle shows a high degree of similarity (35%) to human Nopp140 (NOLC1) (Wise et al., 1997; Isaac et al., 2000) that is also highly phosphorylated by CK2 (Thomas Meier and Blobel, 1992). However, in contrast to Nopp140, Treacle has not been shown to shuttle between the nucleus and the cytoplasm and it does not localize to Cajal bodies (Isaac et al., 2000). The CK2 phosphorylation sites in Treacle mediate its interaction with the E3 ligase CUL3 and its substrate adaptor KBTBD8 that induces monoubiquitylation (Werner et al., 2018).

Treacle is found in the nucleolus in interphase (Figure 1B) and remains associated with nucleolar organizer regions (NORs) during mitosis (Valdez et al., 2004). Treacle is bound to the rDNA promoter by a mechanism dependent on its C-terminal region (Figure 1A) (Gonzales et al., 2005; Lin and Yeh, 2009). The C-terminus is also responsible for its interaction with Upstream Binding Factor (UBF), a component of the Pol I transcription complex (Valdez et al., 2004; Lin and Yeh, 2009). The central part of Treacle (amino acids 526–953) mediates Pol I binding (Lin and Yeh, 2009).

### 2.3 Treacle Promotes Ribosome Biogenesis Through rRNA Transcription and Processing

**FIGURE 2:**
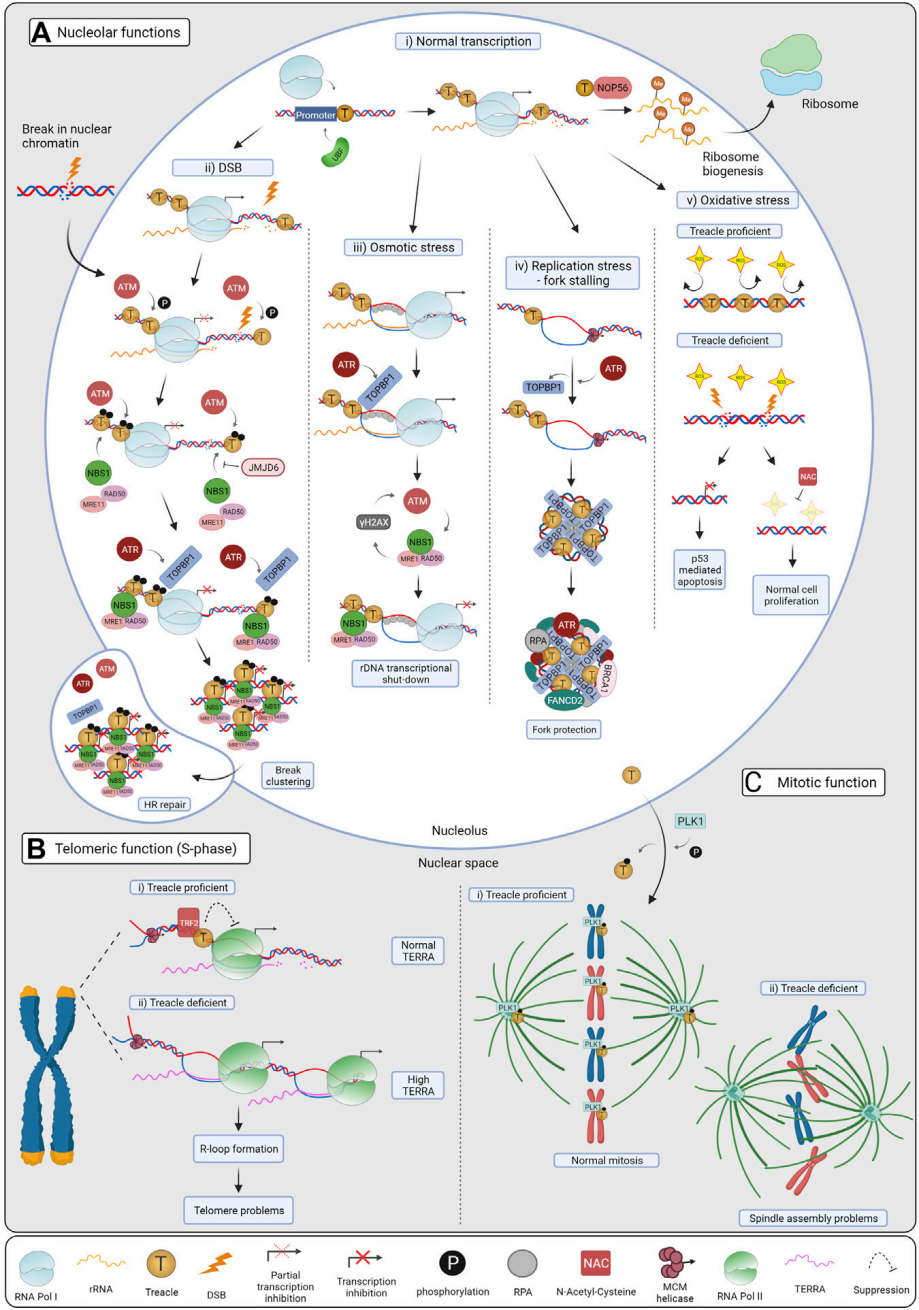
Cellular functions of Treacle. **(A)** Treacle nucleolar functions: i) Normal transcription. Treacle promotes rRNA transcription by recruiting Pol I and UBF to the rDNA promoter. By interacting with NOP56, it also mediates rRNA methylation, essential for mature rRNAs and ribosome production. ii) DSB repair. Upon DSB induction ATM becomes activated and phosphorylates Treacle, leading to Pol I transcriptional inhibition. Treacle in turn recruits the MRN complex and TOPBP1, which then activates ATR. ATR activation is required for complete rDNA transcriptional shut-down, it promotes break clustering, and their eventual translocation to nucleolar caps. In the caps, breaks become accessible to HR factors and repaired. iii) Osmotic stress. Hypoosmotic stress leads to the stabilization of nucleolar R-loops, recruiting RPA and TOPBP1. Treacle is indispensable for TOPBP1 recruitment, and subsequent ATR activation. ATR is the main kinase controlling the response, but ATM activation and NBS1 were also required for Pol I inhibition. iv) Replication stress (RS). RS leads to fork stalling in the rDNA, and activates ATR, which in turn mediates TOPBP1 recruitment through Treacle. TOPBP1 and Treacle form large foci, and promote the recruitment of RS factors, such as RPA, BRCA1 and FANCD2 in the nucleoli. By these means, stalled forks are protected, and RS-mediated rDNA breaks are minimized. v) Oxidative stress. Neuroepithelial cells have high levels of ROS, harmful to the DNA. The rDNA is normally protected by Treacle, however, upon Treacle deficiency, high levels of ROS lead to DSB formation, and consequent rRNA transcriptional inhibition. Nucleolar stress leads to the stabilization of p53 and apoptosis, causing craniofacial defects in TCS patients. When Treacle deficient cells are treated with the ROS scavenger NAC, less DNA damage and apoptosis are observed and craniofacial features are preserved in mice. **(B)** Treacle telomeric functions. i) Treacle is recruited to the telomeric repeats by TRF2, and it controls TERRA transcription in S-phase. Treacle interacts with Pol II and regulates its transcription. ii) TERRA levels increase upon Treacle depletion, leading to R-loop formation and telomere problems. When Treacle is absent, more Pol II associates with the telomeres, and accelerates TERRA transcription. **(C)** Treacle mitotic function. i) Treacle is phosphorylated by PLK1 and localizes to the kinetochores and centrosomes, promoting normal mitosis. ii) In the absence of Treacle, cells have problems with mitotic spindle assembly, chromosome alignment and mislocalization of PLK1. Created with BioRender.com.

Treacle is directly involved in rRNA transcription (Valdez et al., 2004; Lin and Yeh, 2009) and its depletion leads to decreased rRNA levels, both in cultured human cell lines and in heterozygous *tcof1* ^+/−^ mouse embryos (Valdez et al., 2004). It directly binds to the rDNA promoter, and it facilitates pre-initiation complex assembly by recruiting Pol I and UBF (Figures 1B, 2A, panel i) (Valdez et al., 2004; Lin and Yeh, 2009). Treacle downregulation impairs the nucleolar localization of UBF and Pol I, indicating that Treacle functions as a scaffold to maintain the Pol I transcription complex in the nucleolus and thereby facilitates rRNA transcription (Lin and Yeh, 2009).

Treacle also participates in pre-rRNA processing by recruiting NOP56 (Figure 2A, panel i) (Gonzales et al., 2005). NOP56 and fibrillarin, components of the Box C/D snoRNA complex, induce 2’-O-methylation at specific residues of the pre-rRNA. Reduced levels of 2’-O-methylation in rRNA were observed in *Xenopus laevis* oocytes depleted of Treacle and in *tcof1* ^+/−^ mouse embryos (Hayano et al., 2003; Gonzales et al., 2005).

## 3 Treacle is a Central Regulator of Nucleolar Stress Responses

In recent years, an increasing amount of evidence has documented the role of Treacle as a central regulator of the nucleolar response to DNA damage. In the context of DNA damage, Treacle functions both as a regulator of rDNA transcription and as an adaptor protein, facilitating signaling branches activated in response to various genotoxic insults. In the following section, we will discuss the role of Treacle in the response to DNA damage with primary emphasis on DSBs.

### 3.1 Treacle Regulates Nucleolar Transcription in Response to Double-Strand Breaks

The first study that documented a regulation of nucleolar activity in response to DNA damage was published in 2007 by Kruhlak and colleagues (Kruhlak et al., 2007). In mouse embryonic fibroblasts, exposure to γ-irradiation (IR) induced a transient dose-dependent downregulation of rRNA transcription, accompanied by the segregation of UBF into nucleolar caps, characteristic of a transcriptionally inactive nucleolus (Kruhlak et al., 2007). When individual nucleoli were targeted with laser micro-irradiation, a local inhibition of Pol I transcription was observed, indicating that Pol I inhibition occurs *in cis*, proximal to the DSBs (Kruhlak et al., 2007). The inhibition of Pol I was found to be dependent on the well-characterized DDR proteins: ATM, MDC1, and NBS1, preventing Pol I initiation complex assembly and leading to the gradual displacement of elongating Pol I from rDNA distal to the break (Kruhlak et al., 2007).

A later study in human cells by Larsen and colleagues described a second mode of Pol I regulation in response to DNA damage: a trans-compartmental/global Treacle-dependent inhibition of Pol I transcription (Larsen et al., 2014). Damage induced in nuclear chromatin with laser micro-irradiation led to transient repression of rRNA transcription in all nucleoli of the cell. This *in trans* regulation was also dependent on ATM and NBS1, but not MDC1. In both studies, rRNA transcriptional repression was associated with focal accumulation of NBS1 in nucleoli (Kruhlak et al., 2007; Larsen et al., 2014). NBS1 could be recruited to nucleoli independently of MDC1, contrasting the recruitment mechanism of NBS1 to DSBs elsewhere in the genome. However, nucleolar accumulation of NBS1 was orchestrated through the N-terminal FHA/BRCT region that also facilitates its interaction with other phospho-proteins, such as MDC1 (Larsen et al., 2014).

Mass spectrometry analysis of NBS1-associated proteins identified Treacle as the nucleolar interaction partner of the NBS1 FHA/BRCT-domains (Larsen et al., 2014). Treacle, similarly to MDC1, is phosphorylated by CK2 (Isaac et al., 2000), and its interaction with NBS1 was dependent on phosphorylation of Thr210 (Larsen et al., 2014). Importantly, depletion of Treacle led to the reduction of rRNA transcription to a similar level as after IR, and interestingly, no additive effects were observed when IR was combined with Treacle depletion. This data suggest that Treacle is a key component of the pathway that regulates Pol I transcriptional inhibition after DNA damage *in trans*. Treacle depletion also abolished DSB-induced recruitment of NBS1 to nucleoli. Furthermore, forced accumulation of NBS1 in the nucleolus reduced nucleolar transcription levels, suggesting that the Treacle-NBS1 interaction is the functional module regulating rRNA transcription in response to DSBs (Larsen et al., 2014).

The mechanism of Treacle-dependent rRNA silencing, however, remains elusive. It has been proposed that Treacle facilitates nucleolar transcriptional repression indirectly through NBS1. It is likely that Treacle-mediated NBS1 accumulation in the nucleolus leads to increased ATM activation, and phosphorylation of nearby factors participating in transcription (Larsen and Stucki, 2016). In support of this, several proteins regulating rRNA transcription have been identified as ATM targets, including Treacle itself, Pol I subunit RPA34, promoter selectivity factor TAFC1/SL1 and Transcription Termination Factor 1 (TTF1) that are facilitating rRNA transcription (Matsuoka et al., 2007). Phosphorylation of these proteins could alter their interaction with rDNA, and lead to reduced rRNA transcription. Similar phosphorylation-mediated regulation of Pol I transcription has been reported, involving key signaling cascades, such as PI3K/AKT/mTOR and the MAPK/ERK pathways (Grummt, 2003; Morovicz et al., 2021). Upon different external stimuli, these kinases are responsible for phosphorylating members of the Pol I holo-complex, such as SL1 components, TTF1 or UBF, thereby leading to stimulation/inhibition of rRNA transcription (Stefanovsky et al., 2001; Zhao et al., 2003; Mayer et al., 2004; Nguyen and Mitchell, 2013). A similar regulation mechanism could be envisioned in the case of ATM activity in the nucleolus upon rDNA breaks.

Another possibility is a direct involvement of Treacle in rDNA silencing, since its depletion leads to rRNA transcription inhibition. Upon rDNA breaks, phosphorylation of Treacle could potentially weaken its interaction with UBF and Pol I, and thereby fail to support Pol I initiation complex assembly. This could facilitate a gradual displacement of elongating Pol I from rDNA distal to the break, as suggested by Kruhlak and colleagues (Kruhlak et al., 2007).

Furthermore, Treacle might affect rRNA transcription through chromatin remodelers. Upon nucleolar DSB induction, Treacle was found to interact with the histone deacetylase HDAC1 and the histone-arginine methyltransferase CARM1 in mouse embryonic fibroblasts (Franek et al., 2016). HDAC1 is part of the nucleolar remodeling complex, NoRC, and it is important for rDNA silencing (Zhou et al., 2002; Santoro and Grummt, 2005). CRAM1 mediates the deposition of H3R17me2a activating histone modification, facilitating the recruitment of Polymerase-Associated Factor 1C that supports rRNA transcription (Zhang et al., 2010; Wu and Xu, 2012). By interacting with these factors, Treacle could potentially influence the epigenetic status of the rDNA and thereby control transcription.

### 3.2 The ATM-Treacle-NBS1 Signaling Pathway Responds to rDNA DSBs

The tailored nature of the n-DDR also includes specialized repair mechanisms with Treacle placed as the key coordinator of these processes. Nucleolar DSBs lead to transcriptional shut-down and accumulation of proteins in so-called foci. This is followed by nucleolar reorganization and translocation of the rDNA breaks to nucleolar caps (Figure 2A, panel ii) (Harding et al., 2015; van Sluis and McStay, 2017). Repair of DSBs in the nucleolus is compartmentalized; breaks in the nucleolar interior are predominantly repaired by the rapid DNA repair pathway, Non-Homologous End Joining, involving minimal processing of the DNA ends and with limited perturbations of rRNA transcription (Harding et al., 2015; Warmerdam et al., 2016). Persistent breaks, on the contrary, inhibit transcription, cluster and mobilize to the nucleolar periphery, where they are repaired by the Homologous Recombination (HR) repair pathway, utilizing homologous sequences as templates for accurate restoration of the sequence (Warmerdam et al., 2016; van Sluis and McStay, 2017; Korsholm et al., 2019; Marnef et al., 2019). Such segregation of the nucleolus physically separates rDNA originating from different NORs and has been suggested to prevent inter-chromosomal recombination of the identical rDNA repeats (Figure 2A, panel ii).

How rDNA DSBs are recognized and processed in nucleolar chromatin has been a long-standing question. In 2015, two significant studies found ATM to be the predominant kinase in the n-DDR (Harding et al., 2015; van Sluis et al., 2015), similarly to its function elsewhere in the genome. The downstream steps in the n-DDR cascade are however distinct, with the ATM-Treacle-NBS1 axis being a major coordinator of nucleolar responses (Kruhlak et al., 2007; Ciccia et al., 2014; Larsen et al., 2014). ATM-mediated phosphorylation of Treacle initiates the n-DDR and drives repair factor recruitment. This has been observed upon genotoxic stress such as IR and cisplatin, or after targeted induction of rDNA breaks (CRISPR/Cas9, I-PpoI site-specific nucleases) (Ciccia et al., 2014; Korsholm et al., 2019; Mooser et al., 2020). Treacle contains 17 SQ/TQ sites that are putative ATM/ATR phosphorylation substrates (Figure 1), but the relevance of individual sites in the n-DDR has not been easy to determine. The S1199 site of Treacle was initially identified as required for recruitment of NBS1 after IR (Ciccia et al., 2014). Another study, however, only reported a decreased interaction between Treacle and NBS1 after mutation of S1199 (Korsholm et al., 2019). Mutation of all 17 SQ/TQ sites did, however, completely abrogate NBS1 recruitment after targeted rDNA break induction, indicating that several sites are involved (Korsholm et al., 2019). Consistently, after ATM inhibition, no NBS1 accumulation was observed in the nucleoli, and the n-DDR was abrogated, supporting ATM-phosphorylation of Treacle being a requirment for NBS1 recruitment.

Furthermore, the interaction of Treacle with NBS1 after DSBs also required its CK2-phosphorylated SDT domain (Figure 1A) and the FHA/BRCT domain of NBS1 (Larsen et al., 2014; Mooser et al., 2020). Depletion of Treacle or the disruption of either the SDT or the FHA/BRCT domain prevented NBS1 nucleolar accumulation and impaired the n-DDR pathway (Mooser et al., 2020). This data suggests that phosphorylation of Treacle SQ/TQ sites by ATM and the CK2-phosphorylated SDT domain are responsible for initiating the n-DDR by recruiting NBS1 to the nucleolus through its FHA/BRCT domain (Ciccia et al., 2014; Korsholm et al., 2019; Mooser et al., 2020). Furthermore, these findings show similarities in the Treacle-NBS1 binding mechanism facilitating transcriptional inhibition and rDNA repair.

Accumulation of NBS1 could promote further ATM activation at rDNA DSBs, propagating Treacle phosphorylation, and thereby allowing NBS1 and ATM to spread along the rDNA through a feed-forward loop resembling that of DSBs in nuclear chromatin. In the canonical DDR, activated ATM locally phosphorylates H2AX, which in turn recruits the mediator MDC1, leading to NBS1 accumulation, and thereby triggering a feed-forward loop where NBS1 anchors more ATM to the DSB site (Stucki and Jackson, 2006; Chapman and Jackson, 2008). MDC1, however, does not accumulate in the nucleolus, and γH2AX is also less abundant. Treacle would therefore serve both as an ATM target and act as a docking site for NBS1 (Korsholm et al., 2019), likely making it the key mediator protein in the n-DDR, substituting MDC1 function in nucleolar chromatin.

The interaction of NBS1 and Treacle can be modulated by the Treacle-binding histone demethylase JMJD6 (Figure 2A, panel ii). Depletion of JMJD6 led to increased transcriptional suppression and interaction of Treacle and NBS1 measured by proximity ligation assay. However, under the same conditions, NBS1 recruitment to nucleoli after IR was reduced and downstream cap formation was impaired (Fages et al., 2020). Fages et al. speculated that two pools of nucleolar NBS1 may exist: one pool that interacts with Treacle and mediates transcriptional repression, while the other plays a role in the context of the MRN complex, facilitating downstream processes leading to nucleolar cap formation (Fages et al., 2020). Further experimental evidence is, however, required to understand the role of JMJD6 in modification of nucleolar chromatin and rDNA repair.

The interaction between NBS1 and Treacle was also shown to be targeted upon viral infections. The matrix protein of Hendra and Nipah virus binds Treacle and inhibits nucleolar transcription, mimicking the initial steps of the n-DDR. However, binding of the viral proteins prevent the interaction between Treacle and NBS1, possibly compromising the downstream signaling response that would normally be initiated by compromised nucleolar transcription. The binding of Treacle leads to increased viral production, but the reason why viruses inhibit rRNA transcription remains unclear (Rawlinson et al., 2018).

### 3.3 Treacle Recruits the MRN-Complex and TOPBP1 to Activate ATR

NBS1 is known to function as part of the MRN (MRE11-RAD50-NBS1) complex in the canonical DDR. In the nucleolus, however, initial reports could only detect NBS1 in complex with Treacle (Ciccia et al., 2014; Larsen et al., 2014), raising the question of whether NBS1 could function outside the complex. Mass spectrometry analysis had, however, detected MRE11 in purified nucleoli (Andersen et al., 2005) suggesting the presence of the MRN-complex in nucleoli. In a study from 2019, it was convincingly demonstrated that upon rDNA DSBs, NBS1 is recruited to nucleoli by Treacle as part of the MRN-complex and that this facilitates the initial steps of DSB repair (Figure 2A, panel ii) (Korsholm et al., 2019). The MRE11 endonuclease is responsible for the initial processing of the broken DNA ends and in nucleoli MRE11 is required for movement of the rDNA to nucleolar caps (Korsholm et al., 2019). Interestingly, the lack of nucleolar caps upon MRE11 depletion correlates with maintained nucleolar transcription and the other DNA damage kinase, ATR, was found to be required downstream of the MRN-complex for full inhibition of rRNA transcription after DSBs (Figure 2A, panel ii) (Korsholm et al., 2019; Mooser et al., 2020).

Further investigations revealed an additional role of Treacle in the activation of the downstream signaling pathway driven by ATR. The ATR activator TOPBP1, overexpression of which has been associated with ATR-dependent Pol I inhibition and nucleolar segregation (Sokka et al., 2015), accumulates in the nucleolus and co-localizes with Treacle upon rDNA breaks. Moreover, Treacle depletion impaired TOPBP1 recruitment to nucleoli (Mooser et al., 2020) and further characterization of the interaction revealed a direct binding mechanism involving BRCT domain 1, 2, and 5 in TOPBP1 (Mooser et al., 2020). In the C-terminal region of Treacle, several phosphorylation sites were identified that resemble those in known interactors of the TOPBP1 BRCT 0-2 domains, and these sites were demonstrated to specifically facilitate the TOPBP1-Treacle interaction (Figure 1A). TOPBP1 accumulation in the nucleolus after DSBs was then suggested to mediate ATR activation and, in agreement, depletion of TOPBP1 abrogated ATR activation and compromised nucleolar transcription inhibition in response to DSBs (Mooser et al., 2020).

### 3.4 Treacle Promotes rDNA Stability and Resistance to Double-Strand Breaks

The importance of Treacle in the n-DDR is also evident from studies assessing cell viability after genotoxic insults. Depletion of Treacle led to increased platinum sensitivity, indicating a decreased capacity of the n-DDR (Ciccia et al., 2014). Furthermore, upon the induction of targeted rDNA breaks, Treacle depletion led to elevated levels of micronucleation, apoptosis and cell death, suggesting that Treacle is essential for the maintenance of genomic integrity and cell survival (Korsholm et al., 2019). Depletion of Treacle interacting proteins, NBS1, JMJD6, and TOPBP1, also compromised cell viability in response to rDNA breaks (Fages et al., 2020; Mooser et al., 2020), further supporting the role of Treacle as a central coordinator of the n-DDR.

### 3.5 Treacle Regulates the Nucleolar Response to Osmotic Stress

Osmotic stress can lead to DNA damage and recent evidence proposes an important role for Treacle in the nucleolar response to such processes (Velichko et al., 2019). Rapid changes in the concentration of solute molecules around a cell can lead to changes in the cell’s properties, such as water and inorganic ion movement across the cell membrane. This phenomenon is known as osmotic stress and can disrupt canonical cell functions resulting in transcriptional inhibition (Rosa-Mercado et al., 2021), translational blockage (Uesono and Toh-E, 2002) and DNA damage (Dmitrieva and Burg, 2007).

To investigate how nucleoli respond to osmotic stress Velichko et al. (2019) cultured cells under mild hypoosmotic conditions. Treacle was shown to be indispensable for the nucleolar response to this type of stress by facilitating TOPBP1 recruitment and retention in the nucleolus (Figure 2A, panel iii) (Velichko et al., 2019). The authors found that such stress led to increased levels of γH2AX in nucleoli and accumulation of DDR factors, including TOPBP1, RPA and NBS1, initially within the nucleolus and later at the nucleolar periphery (Velichko et al., 2019). Interestingly, ATR was the main kinase coordinating the response to hypoosmotic stress and subsequently ATM was activated, in contrast to the nucleolar response to DSBs (Korsholm et al., 2019). The activated nucleolar DDR led to repression of nucleolar transcription, which was dependent on ATR activation, and to a lesser extent on ATM (Velichko et al., 2019). Velichko et al. demonstrated that ATR activation is facilitated by Treacle and its retention of TOPBP1 in nucleoli (Velichko et al., 2019), similarly to the response to DSBs. In addition, Treacle knockdown cells exhibited compromised silencing of Pol I transcription, while ATM/ATR and downstream DDR cascade was completely abrogated, establishing Treacle as a key upstream factor of the response (Velichko et al., 2019). Hypoosmotic stress has also been shown to regulate nucleolar transcription through lncRNA induced nucleosome repositioning in a Treacle-independent manner (Zhao et al., 2016).

Velichko et al. (2019) further investigated if osmotic stress leads to DNA DSBs. However, they concluded that under hypoosmotic stress the DDR was activated in response to stabilized R-loops, a three-strand nucleic acid structure consisting of two DNA and one RNA strand, including ssDNA stretches coated with RPA. R-loops, detected by immunofluorescence using the S9.6 antibody, formed specifically in the nucleoli. Furthermore they were dependent on Pol I, but not Pol II, activity, suggesting that R-loop formation occurs in transcribed rDNA. Removal of the R-loop structures with overexpression of Ribonuclease H (RNase H), which dissolves the RNA:DNA hybrids, led to complete abolition of the DDR upon hypoosmotic stress, suggesting that ATR is being activated in response to R-loops and initiates the downstream DDR pathway (Velichko et al., 2019).

### 3.6 Treacle Facilitates the Nucleolar Response to Replication Stress

Replication stress (RS) is a driver of DNA damage, and a role for Treacle is also emerging in this context, specifically in nucleolar chromatin. RS generally occurs when the replication machinery encounters barriers interfering with its progression, causing fork slowing or stalling and, if not properly resolved, leading to replication fork collapse and generation of DNA damage and genome instability (Lin and Pasero, 2012). ssDNA, stabilized by the protein RPA, accumulates as a result of fork stalling and facilitates recruitment of proteins, including TOPBP1, resulting in activation of the DNA damage kinase ATR and its downstream effector CHK1. This response helps to stabilize replication forks, prevents origin firing and induces a transient checkpoint response (Blackford and Jackson, 2017).

Obstacles causing RS include secondary DNA structures, DNA bound proteins, DNA lesions, and the transcription machinery, with the latter being the most severe and such encounters leading to “transcription-replication conflicts” (Lin and Pasero, 2012). Conflicts can either be co-directionally or head-on, with co-directional encounters being the most common across the genome due to overlap of origin firing and transcriptional start sites (Lalonde et al., 2021). However, head-on collisions are more likely to induce formation of R-loops, activate the ATR-CHK1 signaling response, and genome instability. In contrast, co-directional conflicts induced the ATM kinase (Hamperl et al., 2017).

The rDNA is particularly susceptible to transcription-replication conflicts due to its high levels of transcriptional activity (García-Muse and Aguilera, 2016). Protective mechanisms therefore exist safeguarding the rDNA from collisions, including polar and bidirectional replication fork barriers (Wang et al., 2013; Akamatsu and Kobayashi, 2015) and temporal/spatial separation of the two machineries (Dimitrova, 2011; Smirnov et al., 2014). More specifically, it was shown that the rDNA replicates in a biphasic manner, where transcriptionally active rDNA replicates during early S phase, while silent rDNA replicates in late S phase (Li et al., 2005). In early S-phase there is clear separation between replication, localized at the periphery of the nucleolus, and transcription that is localized at the nucleolar interior (Dimitrova, 2011). In contrast, the silent rDNA genes maintain their localization and are mostly found in replication foci in the nucleolar interior, adjacent to transcription foci, while replicating in mid and late S-phase (Dimitrova, 2011).

In spite of a structural organization that limits the interaction between transcription and replication, collisions must be processed. However, until recently the nucleolar RS response was unexplored and its unique features are therefore only starting to emerge (Velichko et al., 2021). In order to gain insight into the nucleolar RS response, Velichko and colleagues treated cells with the RS-inducing agents, aphidicolin or hydroxyurea (Velichko et al., 2021). Treatment with either aphidicolin or hydroxyurea showed accumulation of known RS response factors (ATR, CHK1, TOPBP1, RPA, BRCA1, and FANCD2) in the nucleolar interior (Velichko et al., 2021). ATR was found to drive the signaling response to RS, as elsewhere in the genome, and depletion or inhibition of ATR led to complete abrogation of TOPBP1 recruitment in the nucleolus (Velichko et al., 2021). The nucleolar interaction partner of TOPBP1 was shown to be Treacle, necessary for TOPBP1 nucleolar retention and TOPBP1-mediated ATR activation upon RS (Figure 2A, Panel iv) (Velichko et al., 2021). ATR activity was therefore required for the initiation of the nucleolar RS response, but Treacle-TOPBP1 is crucial for its reinforcement. Again, Treacle’s role in TOPBP1 recruitment and downstream DDR activation was specific to nucleoli and not required for RS responses in nuclear chromatin (Velichko et al., 2019).

Velichko et al. (2021) also mapped the Treacle-TOPBP1 interaction in more detail. In addition to the previously described interaction sites, the BRCT domains 1, 2, and 5 in TOPBP1 (Mooser et al., 2020), it was found that deletion of TOPBP1’s ATR activating domain (AAD) gives a phenotype resembling Treacle or ATR knock-down. This suggests that ATR activation is required for the binding between TOPBP1 and Treacle (Velichko et al., 2021). In contrast, deletion of BRCT 7 or 8 did not disrupt the interaction, but resulted in a minor reduction in ATR activation and mild nucleolar transcriptional repression (Velichko et al., 2021). Interestingly, BRCT 7 and 8 are involved in oligomerization and the accumulation of TOPBP1 in nucleoli resulted in formation of few very large spherical foci surrounding the FC-compartment. Furthermore, Treacle and TOPBP1 become resistant to salt extraction upon RS, suggesting that the complex may form a macromolecular scaffold structure in nucleoli (Figure 2A, panel iv) (Velichko et al., 2021).

Surprisingly, nucleoli have been found to be very resistant to RS. Neither significant nucleolar transcriptional silencing nor nucleolar cap formation was observed under RS, suggesting that there might only be local transcriptional inhibition contained in each nucleolus, and that RS can be resolved in the nucleolar interior without rDNA translocation to the nucleolar periphery (Velichko et al., 2021). In agreement, RS did not lead to replication fork collapse and generation of DNA DSBs, indicating an intrinsic capability to overcome RS in nucleoli (Velichko et al., 2021). These findings agree with previous results demonstrating that co-directional transcription-replication conflicts are well tolerated by cells and do not cause DSBs to the same extent as head-on collisions (Hamperl et al., 2017). However, in the absence of Treacle or TOPBP1, the ATR checkpoint and recruitment of RS response factors (BRCA1, FANCD2) were completely abrogated and transcriptional shutdown was observed. Under such conditions cells were unable to recover from the stress, even if it was transient and DSBs were observed (Velichko et al., 2021). These novel findings are of utmost interest as they demonstrate how the ability of cells to overcome co-directional collisions in rDNA is highly dependent on Treacle. The role of Treacle in rRNA transcription could augment the phenotype, as compromised transcription influences the nature of collisions between the transcription and replication machineries. In *E. coli* co-directional collisions have been shown to induce genome instability specifically when transcription is fully arrested and RNA polymerase backtracking occurs (Dutta et al., 2011). Further studies are therefore required to understand how Treacle promotes nucleolar resistance to replication stress and whether its roles both in stimulation of transcription and in DNA damage signaling are important. In addition the signaling pathways activated upon replication stress, in the absence of Treacle or TOPBP1, remain to be characterized.

Finally, the study by Velichko et al. (2021) showed a recruitment of DNA repair factors into nucleoli upon RS, including ATR, TOPBP1, RPA, BRCA1, and FANCD2. It has been widely accepted in the field that the damaged rDNA translocate to the nucleolar periphery to form caps in order to become accessible to the repair factors that cannot accumulate in the nucleolus. Interestingly, in this study Velichko et al. (2021) showed that upon RS there was no cap formation and instead, the repair factors could be retained in the nucleolar interior accumulating around Treacle-TOPBP1 intranucleolar foci (Figure 2A, panel iv). These findings demonstrate that access to the nucleolus is dynamically regulated and the interaction between damaged rDNA and nuclear repair factors can be facilitated by other means than nucleolar cap formation.

In conclusion, the data from this study underline the significance of the Treacle and TOPBP1 interaction to activate ATR and act as a scaffold platform for RS response factors in nucleoli.

### 3.7 Treacle Safeguards Against Oxidative Damage

Oxidative damage poses another threat to cell fidelity and arises when reactive oxygen species (ROS) are in excess and react with lipids, proteins, or DNA, giving rise to a cellular state referred to as oxidative stress (Bedard and Krause, 2007; Pizzino et al., 2017; Moloney and Cotter, 2018). DNA lesions induced by ROS include base-lesions and DNA strand breaks that can cause alterations in the DNA unless resolved (Pizzino et al., 2017; Moloney and Cotter, 2018).

Treacle was first associated with oxidative damage in a mass spectrometry screen and was identified as a protein that was significantly downregulated in response to the oxidant H2O2 in A549 lung cells (Figure 2A, panel v) (Duan et al., 2010). Furthermore, depletion of Treacle made the A549 cells more sensitive to oxidative stress causing decreased cell viability (Duan et al., 2010). Treacle was found to be degraded in a dose-dependent manner typical for an oxidant-absorbing protein, and it was proposed that Treacle functions as a p53-independent regulator of oxidative damage (Duan et al., 2010).

## 4 Nuclear Functions of Treacle

### 4.1 Treacle Protects Telomeres by Regulation of Telomeric Repeat–Containing RNA Transcription

Although mostly found in the nucleolus, Treacle also localizes to the nucleoplasm during S-phase. It is specifically recruited to the telomeres, where it plays a critical role in maintenance of telomere integrity (Nie et al., 2021). Telomeres are composed of arrays of 5’-TTAGGG-3’ repeats that protect the end of the chromosomes (de Lange, 2018). These sequences are transcribed into Telomeric repeat-containing RNA (called TERRA) (Azzalin et al., 2007). The repetitive nature of telomeres and transcription of TERRA can interfere with telomere replication during S-phase. In agreement, TERRA levels are decreased during S-phase in proliferating cells (Porro et al., 2010). This is mainly due to the formation of R-loops that can impede the progression of the replication fork (Maestroni et al., 2017). Defects in telomere replication cause a fragile telomere phenomenon characterized by telomere-free chromosome ends and multiple telomere signals, among others. For these reasons, TERRA levels need to be tightly regulated during S-phase, and Treacle was shown to play a role in this process (Figure 2B). Nie et al. (2021) detected Treacle foci outside the nucleoli in U2OS cells colocalizing with telomeres. Treacle has previously been found to interact with TRF2 (telomeric repeat-binding factor 2), a component of the shelterin complex (Giannone et al., 2010), and Treacle’s recruitment to telomeres was consistently dependent on TRF2. The interaction between Treacle and TRF2 occurs mainly during S-phase and via the C-terminal domain of Treacle (Figure 1A). In the absence of TRF2, Treacle is not recruited to telomeres (Figure 2B) (Nie et al., 2021).

RNA Polymerase II (Pol II) transcribes the telomeres into TERRA, which in turn regulates the activity of telomerase (Schoeftner and Blasco, 2008). As shown by Nie et al. (2021), Treacle interacts with Pol II through its repeated domains and mainly during S-phase. When Treacle is depleted, there is an increased transcription of TERRA and association of active Pol II with telomeres. Elongating Pol II levels at telomeres significantly dropped when full-length Treacle was expressed, indicating that Treacle interacts with Pol II to suppress its transcription activity (Figures 1A, 2B).

Elevated levels of TERRA are associated with R-loop formation, and indeed, Treacle depletion causes increased R-loop formation which in turn blocks progression of replication forks (shown through increased PCNA and RPA, markers of fork stalling) (Nie et al., 2021). The absence of Treacle also leads to fragile telomere phenomena due to replication defects, increased DDR at telomeres, and genome instability. These defects are rescued by masking TERRA or by overexpression of RNase H1, an R-loop eraser, confirming that they are independent of Treacle’s function in ribosome biogenesis.

In summary, Treacle is essential for the integrity of telomeres by suppressing telomere transcription and R-loop formation, hence avoiding replication fork stalling and DDR activation at telomeres (Nie et al., 2021).

### 4.2 Treacle Regulates Mitotic Spindle Orientation and Cell Cycle Progression

A study by Sakai et al. (2012) used *tcof1* ^+/−^ heterozygous mice to assess the importance of Treacle in neurogenesis in mammalian brain development and uncovered a novel role for Treacle ensuring proper mitotic spindle orientation and cell cycle progression. Initially, the authors found that Treacle mutant mice show brain hypoplasia, reduced number of neurons in specific cortical layers during neurogenesis, and increased number of ectopically located mitotic cells compared to the control mice (Sakai et al., 2012). In addition, immunostaining assays identified Treacle at the centrosomes, the organizing centers of the spindles, and the kinetochores during mitosis, indicating a direct role of Treacle during mitosis (Figure 2C) (Sakai et al., 2012).

Defects in spindle formation and chromosome alignment were confirmed in HeLa cells depleted of Treacle (Sakai et al., 2012). Interestingly, Treacle showed co-localization with Polo-like kinase 1, (PLK1), a mitotic kinase, and in a Treacle-depleted background PLK1 was dislocated from the kinetochores, indicating that Treacle may act as a scaffold for proper PLK1 localization (Sakai et al., 2012). Co-immunoprecipitation assays showed a mitosis-specific interaction between Treacle and PLK1 (Figure 2C) (Sakai et al., 2012), in agreement with previous data where Treacle was identified as one of PLK1’s interactors by mass spectrometry (Lowery et al., 2007). Additional immunoprecipitations with protein fragments showed that the interaction occurs between the C-terminal Polo-Box domain in PLK1 and the N-terminal domain of Treacle (Figure 1A) (Sakai et al., 2012). Furthermore, *in vitro* and *in vivo* phosphorylation assays confirmed that Treacle is a phosphorylation substrate of PLK1 and Cdk1/CyclinB1 (Sakai et al., 2012). In conclusion, this study unravels a novel role for Treacle during mitosis that influences brain development in mice and may have implications for human diseases.

## 5 The Role of Treacle in Treacher Collins Syndrome

The genetic link of Treacle, POLR1C, POLR1D, and POLR1B to Treacher Collins Syndrome (TCS) in humans strongly indicates that disturbance of ribosome biogenesis is linked to the development of the disease (Dixon et al., 2006; Jones et al., 2008). TCS is characterised by distinct abnormalities of the head and face present already at birth. The structures affected in TCS are primarily derived from neural crest cells, a temporary migratory cell population that gives rise to most of the peripheral nervous system and the craniofacial bone, cartilage and connective tissue. The disease therefore appears when the fitness of the neural crest cells is affected and the formation of tissues is compromised (Dixon et al., 2006).

A heterozygous *tcof1* ^+/–^ mouse model was established and found to exhibit a phenotype that mimics TCS and has therefore been used as an *in vivo* model to study the disease (Dixon et al., 2000). The first connection between Treacle and formation of neural crest cells came from WT mice. In the neuroepithelium and in the craniofacial tissues, Treacle expression is tightly regulated during early embryogenesis, with very high levels at embryonic days 8.5–9.5, whereas it is almost undetectable after embryonic day 10.5 (Dixon et al., 2006). The short window of expression of Treacle overlapped with neural crest cell formation and migration suggesting a possible involvement of Treacle in these processes.

**FIGURE 3:**
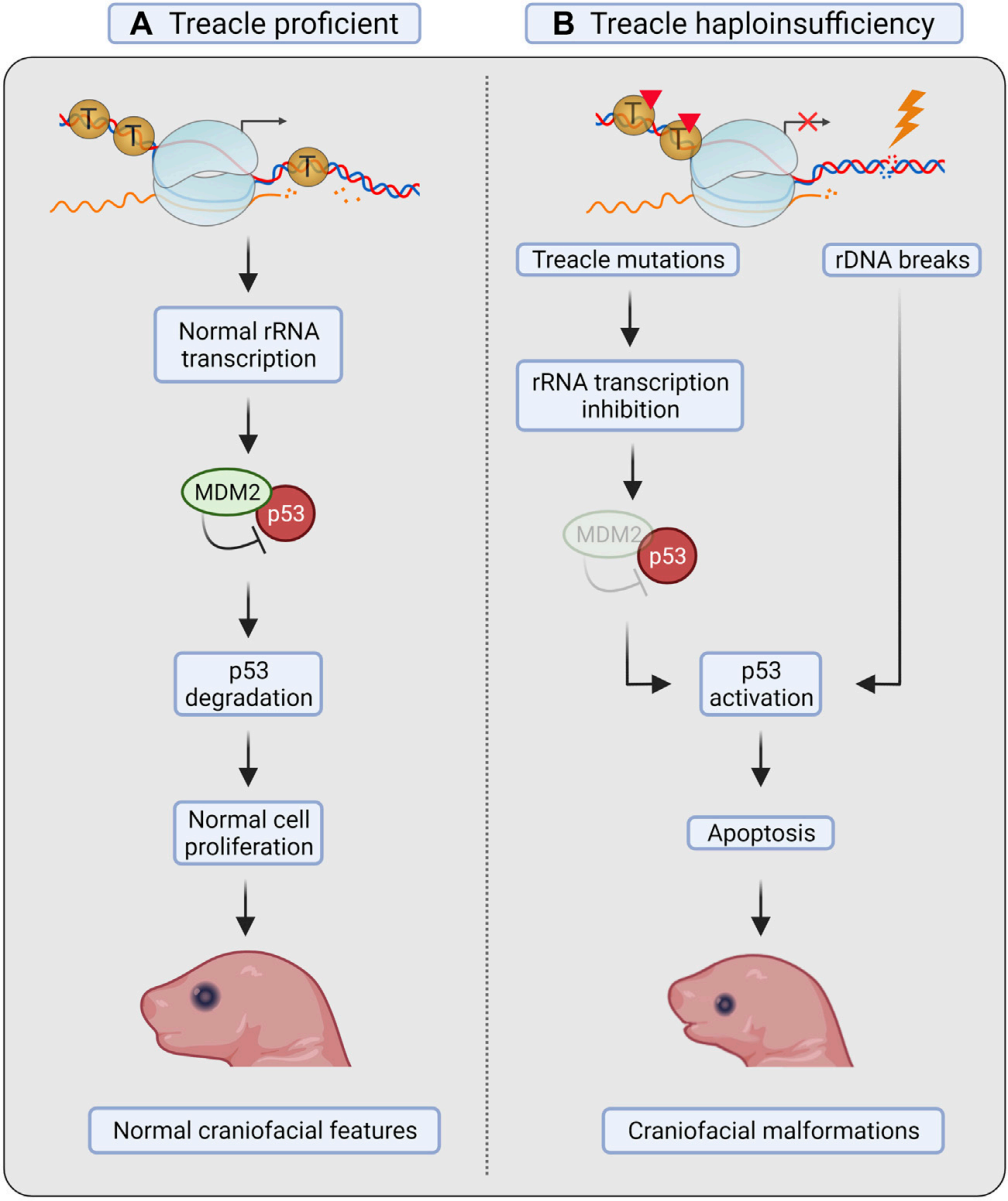
Treacle in Treacher Collins syndrome. **(A)** Treacle proficient mice show normal rRNA transcription, proliferation and apoptosis in neural crest cells (NCC), and develop normal craniofacial features. **(B)** In mice with Treacle haploinsufficiency (*tcof1* ^+/–^), however, increased apoptosis was observed in NCC. Mutations in Treacle compromise rRNA transcription, leading to ribosomal protein-mediated sequestration of MDM2, and consequent p53 stabilization. Increased rDNA damage in the absence of Treacle-mediated repair can also facilitate p53 stabilization. Activation of p53 leads to increased apoptosis of NCC and to the development of craniofacial malformations. Created with BioRender.com.

*tcof1* ^+/–^ mice displayed elevated levels of apoptosis in the neuroepithelium and impaired formation of neural crest cells, leading to incomplete or underdeveloped craniofacial structures (Dixon et al., 2006). The phenotype could be rescued by inactivation of p53 (Jones et al., 2008). Loss of one copy of p53 considerably reduced neuroepithelial apoptosis, whereas loss of both copies of p53 was needed to obtain a phenotype equivalent to that of controls and allow viable pups without TCS-associated defects (Figure 3) (Jones et al., 2008).

Due to the nucleolar localization of Treacle, the 28S rRNA transcription levels were measured. The authors found defects in ribosome biogenesis concurrently with induction of apoptosis in *tcof1* ^+/–^ mice, suggesting that defective ribosome biogenesis results in activation of p53 (Figure 3). Activation of p53 in response to impaired ribosome biogenesis can occur through repression of the ubiquitin ligase MDM2 by the 5RNP complex (composed by the 5S rRNA, and the 60S ribosomal proteins RPL5/uL18 and RPL11/uL5) (Fumagalli et al., 2012; Sloan et al., 2013). Notably, ribosome biogenesis was not restored after p53 inactivation, as shown by the continuous decrease in 28S rRNA levels (Jones et al., 2008). These results therefore point to p53 as the primary downstream effector activated by nucleolar stress in TCS, but the cause of nucleolar stress was not further investigated.

The role of DNA damage in TCS was first reported by Sakai et al. This study showed that the neuroepithelium in mice is found in a high oxidative state, and that Treacle, *via* its function in DNA repair, enables neural crest cells to cope with the high level of ROS (Figure 2A, panel v) (Sakai et al., 2016). It was observed that *tcof1* ^+/–^ embryos showed elevated levels of both DNA damage and apoptosis in neuroepithelial cells compared to control mice (Sakai et al., 2016). It was also demonstrated that the neuroepithelial cells were more sensitive to elevated levels of oxidative stress: treatment of E8.5 embryos with a strong ROS generator led to a considerable increase of apoptotic cells specifically in the anterior neuroepithelium (Sakai et al., 2016).

The endogenous levels of ROS were not elevated in embryos with haploinsufficiency for Treacle (*tcof1* ^+/–^) compared to *tcof1* ^wt^ embryos. Sakai et al. (2016) therefore suggested that haploinsufficiency of Treacle did not influence endogenous ROS levels in the neuroepithelium, but that *tcof1* ^+/–^ caused apoptosis due to a reduced capacity to cope with the oxidative stress (Figure 3) (Sakai et al., 2016).

Sakai et al. (2016) interestingly found that the TCS-associated malformations resulting from Treacle haploinsufficiency can be prevented by treatment with the antioxidant N-acetyl-cystein (NAC). Additionally, experiments with short term (E5.5–E10.5) and long term (E5.5–E17.5) treatment with NAC *in utero* were performed, and especially long-term treatment showed a very encouraging amelioration of the craniofacial phenotypes (Figure 2A, panel v) (Sakai et al., 2016).

Collectively, these results indicate that Treacle’s function as a regulator in the oxidative stress response is important for the development of TCS. Additionally, minimizing ROS-induced DNA damage and subsequent apoptosis of neural crest cells could prevent craniofacial malformations in TCS mice (Sakai et al., 2016).

A second study conducted in zebrafish also concluded that elevated levels of DNA damage could be the cause of TCS (Calo et al., 2018). Interestingly, the authors found that *polr1d^−/−^* and *polr1c^−/−^* zebrafish embryos had elevated levels of γH2AX, similarly to *tcof1* ^+/–^ embryos, and that the level of γH2AX correlates with the severity of the facial malformations. The authors also investigated the ability of rDNA DSBs (induced by the I-PpoI endonuclease) to activate the p53 response and found a direct correlation, whereas induction of DSBs genome-wide (induced by AsiSI) activated p53 to a lesser extent. The authors therefore suggested that transcriptional stress in nucleoli leads to rDNA damage, p53 activation and apoptosis in neural crest cells (Figure 3) (Calo et al., 2018).

Importantly, the authors also demonstrated how loss of a general regulator, such as TCOF1, that compromises nucleolar integrity can lead to tissue-specific phenotypes. The authors abrogated TCOF1 expression in *Xenopus Laevis* embryos by injection of morpholinos and observed craniofacial phenotypes very similar to those in TCS. Upon increased doses of morpholinos, overall growth impairment could be detected, indicating that craniofacial structures are hypersensitive to the loss of TCOF1 (Calo et al., 2018). The authors also induced rDNA damage by injecting I-PpoI mRNA into *Xenopus Laevis* embryos, and again found a predominant effect on the head development. At low doses, the phenotype resembled that of TCS and with increasing doses more severe phenotypes could be detected in the head region and general growth impairment was observed (Calo et al., 2018). These results show how defects in TCOF1 and rDNA damage result in the tissue-selective and dose-dependent phenotypes associated with TCS.

In summary, both the role of Treacle in nucleolar transcription and as a nucleolar DDR factor are important to prevent nucleolar stress during embryogenesis and the development of TCS. Haploinsufficiency of Treacle decreases the fidelity of rDNA transcription, resulting in rDNA damage and p53 activation. The activation of p53 is likely exacerbated by impairment of the broad spectrum of repair mechanisms that is normally promoted by Treacle.

## 6 Treacle in Cancer

Cancer is another pathological condition that has been associated with Treacle. Treacle controls several cellular processes, including ribosome biogenesis, DNA repair, proliferation, apoptosis and differentiation (Dixon et al., 2006; Sakai et al., 2012, 2016; Dai et al., 2016). These pathways have counteracting roles in tumorigenesis, opening the possibility that Treacle acts either as a tumor suppressor or an oncogene. Recent studies are providing the first information towards understanding Treacle in cancer.

Genome instability is a hallmark of cancer, and Treacle deficient cells show accumulation of DNA lesions and increased sensitivity to genotoxic stress, potentially contributing to cancer development (Ciccia et al., 2014; Korsholm et al., 2019). Interestingly however, in contrast to other DNA repair deficiency syndromes, TCS does not show increased cancer predisposition, indicating that Treacle haploinsufficiency is not promoting tumor development. This might be explained by the functional allele present in TCS or by increased apoptosis in cells with reduced levels of Treacle. Treacle impairment furthermore leads to decreased ribosome biogenesis and lower proliferation, potentially having anti-cancer effects (Dixon et al., 2006; Jones et al., 2008).

Treacle upregulation is however emerging as a tumor promoting factor. Elevated Treacle levels correlated with poor prognosis, and shorter survival in different cancer types (Gu et al., 2022; Hu et al., 2022; Wu et al., 2022). This could be attributed to Treacle’s function in facilitating rRNA transcription. Cancer cells are addicted to high levels of ribosome biogenesis, serving their increased proliferation capacity (Derenzini et al., 1998, 2000; Bywater et al., 2012). Moreover, high ribosome biogenesis rates are downregulating p53-dependent apoptotic pathways, further promoting uncontrolled growth (Zhang and Lu, 2009; Donati et al., 2012; Lindström et al., 2018).

How Treacle contributes to cancers, correlates with survival, and may present a future anti-cancer target is further discussed in the perspective article “Treacle is upregulated in cancer and correlates with poor prognosis in kidney and liver cancer” by Oxe and Larsen.

## 7 Concluding Discussion

Evidence of a distinct DNA damage response is emerging in the context of nucleolar chromatin, with Treacle playing a key role. As a unique nucleolar adaptor protein with multiple direct interaction partners, Treacle coordinates several nucleolar responses after DNA damage. It is an integral part of the ATM-driven cascade, being a target of ATM itself, but also facilitating the accumulation of other ATM-targets such as NBS1 and TOPBP1 in foci after DNA damage. Furthermore, Treacle promotes the downstream activation of ATR in nucleoli and thereby a significant restructuring of the nucleolus that allows homology-driven repair in nucleolar caps.

Treacle also inhibits nucleolar transcription after DNA damage but the underlying mechanism remains less clear. Treacle may be directly modified, resulting in a decreased stimulation of transcription, resembling the decreased rRNA transcription scenario observed upon haploinsufficiency in TCS. Alternatively, Treacle may enhance ATM activity locally upon DNA damage and induce transcription inhibition by phosphorylation of other factors facilitating rRNA transcription.

On the contrary, Treacle has been known to promote rRNA transcription, through UBF and Pol I retention, in the context of ribosome biogenesis for nearly two decades, but limited mechanistic understanding has been reached. Efforts to elucidate the mechanisms by which Treacle regulates rRNA transcription may hold the key to bridge the function of Treacle in the nucleolar DNA damage response and in ribosome biogenesis.

Interestingly, the adaptor protein MDC1, viewed as the nuclear paralog to Treacle, was originally identified as a nuclear transcriptional transactivator (Ozaki et al., 2000). However, later studies have focused primarily on the role of MDC1 as an adaptor protein that facilitates accumulation of other factors to sites of DNA damage with limited attention beening paid to its role in regulation of transcription. Important lessons about both the nucleolar and the canonical DDR may therefore be learnt from understanding the role of Treacle (and MDC1) in the regulation of transcription.

Finally, the role of Treacle as a regulator of both rRNA transcription and rDNA damage likely contributes to the development of TCS. The impairment of transcription activates the nucleolar checkpoint and can lead to DNA damage that cannot be efficiently resolved in the absence of Treacle and therefore leads to induction of apoptosis in the craniofacial tissues affected in TCS. Neural crest cells are hypersensitive compared to other tissues and further studies will be needed to clarify if pharmacological intervention is a possibility for TCS patients.

## Glossary

ATM: Ataxia Telangiectasia Mutated
ATR: Ataxia Telangiectasia and Rad3-Related
BRCA1: Breast Cancer Type 1 Susceptibility Protein
CK2: Casein Kinase 2
DDR: DNA Damage Response
DSB: DNA Double-Strand Break
FANCD2: Fanconi Anemia Group D2 Protein
FHA/BRCT: Forkhead-Associated (FHA) and BRCA1 C-Terminal (BRCT)
HR: Homologous Recombination
IR: γ-irradiation
JMJD6: Jumonji C Domain-Containing Protein 6
MDC1: Mediator of DNA Damage Checkpoint 1
MRE11: Meiotic Recombination 11 Homolog A
NAC: N-Acetyl-Cystein
NBS1: Nijmegen Breakage Syndrome 1
n-DDR: Nucleolar DNA Damage Response
NLS: Nuclear Localization Signals
NOR: Nucleolar Organizer Region
PLK1: Polo-Like Kinase 1
Pol I: RNA Polymerase I
Pol II: RNA Polymerase II
rDNA: Ribosomal RNA Genes
ROS: Reactive Oxygen Species
RPA: Replication Protein A
rRNA: Ribosomal RNA
RS: Replication Stress
SL1: Selective Factor 1
TCOF1: Ribosome Biogenesis Factor 1
TCS: Treacher Collins Syndrome
TERRA: Telomeric Repeat-Containing RNA
TOPBP1: DNA Topoisomerase II Binding Protein 1
TRF2: Telomeric Repeat-Binding Factor 2
TTF1: Transcription Termination Factor 1
UBF: Upstream Binding Factor
γH2AX: Phosphorylated (γ) H2A Histone Family Member X
